# The Hospital. Nursing Section

**Published:** 1905-08-26

**Authors:** 


### The Hospital.

### Nursing Section.

Contributions for this Section of "The Hospital" should be addressed to the Editor, "The Hospital" Nursing Section, 28 & 29 Southampton Street, Strand, London, W.C.

No. 987.—Vol. XXXVIII. SATURDAY, AUGUST 26, 1905.

### Notes on News from the Nursing World.

### IMPORTANT TO MATRONS.

For some weeks past there have been appearing in the Institutional Section of The Hospital a series of articles on the Matron's Department of the hospital. These articles review all aspects of a matron's duties; those referring to the training of probationers, the distribution of the staff, cost and management, and those relating to the commissariat in general. The information has been gathered from a large number of hospitals, and the author is thoroughly conversant with the subject. These articles will continue to be published regularly, as far as possible, and cannot fail to prove of service to all concerned in the practical management of our hospitals. It is to be hoped that they will prove of sufficient interest to stimulate a useful correspondence on those points upon which varying opinions must exist.

### ARMY NURSING SERVICE RESERVE.

We are desired by her Royal Highness Princess Christian, the President, and the Committee of the Army Nursing Service Reserve to announce that "the Committee of the Army Nursing Service Reserve is anxious to fill up vacancies which have occurred amongst its members consequent on the resignation of a hundred ladies on their marriages, whilst seventy other of the members have been appointed to Queen Alexandra's Imperial Military Nursing Services at home and in India. As the services of the Reserve continue to be in request, the Committee will be glad to receive any applications for membership. The conditions of this Service have recently been revised and the pay increased. Applications for membership should be made to the Honorary Secretary, Army Nursing Service Reserve, 68 Victoria Street, London, S.W., who will forward the regulations and forms of application to candidates."

### THE TESTIMONIAL TO MISS PETER.

The Hon. Secretaries of the Associations of "Queen's" Superintendents of the Northern and Southern Counties ask us to announce that subscriptions to the testimonial to be presented to Miss Peter, the retiring General Superintendent of the Queen Victoria's Jubilee Institute for Nurses, by Queen's nurses, past and present, will be received up to October 7th, when the Fund will be finally closed. Subscriptions should be sent to the Hon. Treasurer of the Fund, Miss Mantell, 54 Knatchbull Road, Camberwell, London, S.E.

### TRAINED NURSES IN TURKEY.

It is about seven years since a well-known American surgeon started in Marsovan, a town in Asia Minor, 60 miles from the Black Sea coast, a little building where the Turkish poor could receive free medical and surgical treatment. The institution has gradually developed into an important hospital, and it is now a training school for nurses, where a number are constantly instructed. At the outset the duties of a nurse were considered only fit for the humblest of domestic servants, but after one native girl of good family was induced to take up the training many more offered their services, and there are at the present time a number eager to enter the school who cannot be accepted until more funds are available. We hope that the money required will be obtained; and, at any rate, it is satisfactory to learn that the natives make excellent nurses and, when carefully trained, accomplish excellent results. The further effort of the founder of the hospital at Marsovan to establish a training-school for nurses at Constantinople itself will be watched with interest and sympathy.

### SCARLET FEVER PATIENTS NURSED BY A WORKHOUSE INMATE.

An extraordinary state of affairs was reported to the Local Government Board by the medical officer at Bideford Workhouse. He informed the Board that there were two cases of scarlet fever in the workhouse, and that the patients were being looked after by an inmate. An explanation being requested, the master said that the cases were very slight, and that the inmates only had charge of the patients for two days, until other arrangements could be made. The plea of the master is quite inadequate. However, the Local Government Board having intimated that on the occurrence of scarlet fever, a nurse should be at once engaged to take charge of the patients, who should be isolated and not left to the care of an inmate, we hope that the master will not again imagine that because cases of scarlet fever are supposed to be "very slight," they may be nursed by entirely untrained persons, ignorant, among other things, of the precautions which should be taken to prevent the spread of one of the most infectious of diseases.

### A REDUCTION OF THE STAFF AT WINDSOR.

At the last meeting of the Windsor Guardians a report of the Infirmary Committee as to making an appointment on the nursing staff was considered. The Committee had selected three candidates to come before the Board, but, in spite of the recommendation of the medical officer of the workhouse that the vacancy should be filled up, all the guardians in attendance voted for an amendment postponing the question for the present on the ground that there are already sufficient nurses for the number of patients in the infirmary. The number of patients in the infirmary is 147, and there were originally nine nurses, who have now been reduced to eight. The decision of the guardians is, in our judgment, a mistake in policy, for in such a case the recommendation of the medical officer should have been regarded as conclusive. If, too, there was any doubt as to filling up the vacancy, the three candidates who presented themselves before the guardians should not have been put to unnecessary trouble.

### HOUSE ARRANGEMENTS AT LIMERICK INFIRMARY.

There has been another acrimonious discussion at the monthly meeting of the Governing Committee of the Limerick County Infirmary. In this instance the controversy was due to a letter written by Dr. Kennedy, one of the visiting staff, who complained that the matron had not notified to him her intention of going away for a week's holiday. Miss Mayne, the matron, stated that when she got her holidays from the Board of Governors she did not think it was necessary she should acquaint the medical staff of her absence, particularly when she had a capable substitute in the person of her staff nurse. In the end the suggestion that a minute should be made rendering it incumbent on the secretary to notify the visiting staff when the house staff were absent, was adopted. This is a reasonable arrangement, but we do not think that there was any cause for the criticisms which were made by some of the speakers, or that there was the slightest intention on the part of the matron to treat the visiting staff with discourtesy.

### THE HOURS OF DUTY AT WANDSWORTH INFIRMARY.

A nurse who gave evidence last week at an inquest on a baby at Wandsworth Poor-law Infirmary was asked by one of the Wandsworth Guardians how long the nurses were on duty. The Coroner took exception to his intervention, and said that an inquest was not a proper occasion to inquire into the working of the infirmary. The Guardian observed that he wanted to show that the nurses work long hours, whereupon the Coroner rejoined that as one of their employers the Guardian had the right to raise such questions in the board-room. That is, of course, the proper place for raising them, and if the hours of the nurses at Wandsworth Infirmary are too long it is the duty of the Guardians to see that they are reduced.

### THE LEGAL RESIDENCE OF THE REGISTERED NURSE.

The State-registered nurse in America has her own peculiar troubles. The superintendent of an American hospital who made up her mind to be registered encountered considerable difficulties in carrying out her intention. The initial condition, involving the filling in of many papers and the attachment of numerous signatures, occupied a long period; and in the meantime the nurse had resigned her position as superintendent and had taken up private nursing, which at the date her papers were sent to Albany, required her presence in a Connecticut town not far from New York. Having explained to the public notary that she was born, educated, and graduated in New York, and wished to practise nursing there, in order to be quite accurate she asked where her legal residence was. He replied, "It is where you have your washing done!" adding, "Well, where you hang up your hat!" The papers were therefore returned with the Connecticut town given as the legal residence. Presently the nurse received an intimation that it would be necessary for her to reside in New York State. Being then engaged on a case in New York, she wrote and requested that the papers might be returned for correction. Next, following another letter stating that her papers were in the hands of the nurse examiners, and, after a long interval, came an intimation that she must claim New York State as her residence. She replied that she was living in New York, and was then told that she must make a legal claim of New York as her residence. Thereupon she went to a second notary, who prepared an affidavit, and advised her to submit it for approval before being sworn. She paid his fee, consulted him again, was sworn, and paid another fee. At last, in despair, she again visited the notary she saw in the first instance and inquired, once more, "Can you tell me where my legal residence is?" His answer this time was prompt. "It is where you husband votes." "And this," adds the nurse, "was the unkindest cut of all, for I have no husband, and never expect to have one." Meanwhile, she still remains in doubt as to the place of her legal residence.

### A QUESTION OF PRINCIPLE.

On the ground that the medical officer of the Carmarthen Workhouse describes the recently-appointed untrained nurse as kind and sympathetic in her treatment of the sick, the Carmarthen Guardians have asked the Local Government Board to reconsider their decision not to sanction the appointment. We hope that the request will not be acceded to. The question involved is not one of persons, but of principle. There are many untrained women who, so far as kindness and sympathy are concerned, are all that could be desired, just as there are some fully-trained nurses without kindness and destitute of sympathy. But the training is essential, and if the Local Government Board consent to dispense with it in one case they will be expected to do so in others.

### THE PRIVATE NURSING DEPARTMENT AT SWANSEA.

It will be recollected that a short time ago the Committee of Management of the Swansea Hospital recommended that the private nursing department should be suspended. At the monthly meeting of the Board, however, the recommendation was opposed at the instance of the honorary medical staff, and Dr. Brook, in expressing his agreement with the staff, urged that double or treble the number of nurses now employed are required. As it was said that the nurses' home was needed for the accommodation of the hospital nursing staff, he suggested that a residence adjoining the hospital should be secured in order to house the private nurses. The recommendation of the Committee was upheld by the speaker, and eventually it was referred back for further consideration. Apparently, there is a great demand in Swansea for private nurses, and it should, of course, be met by sane measures.

### THE TRAINED MATRON AS SUPERINTENDENT NURSE.

We are glad to learn that the Local Government Board have sanctioned the action of the Guardians of Elham Workhouse who have appointed Miss Hillidge, matron of the workhouse, superintendent nurse of the infirmary, with an addition of £5 per annum to her salary. Wherever the workhouse matron is a fully-trained nurse she is a suitable person to undertake the duties of superintendent.

### THE MATRON OF THETFORD COTTAGE HOSPITAL.

An interesting ceremony took place at Thetford Cottage Hospital the other day, when Miss Sculby, who has been matron of the institution since its opening seven years ago was presented with an illuminated address and a purse containing £20. In the address, which was signed by the chairman, treasurer, and honorary secretary, on behalf of the committee, Miss Sculby was assured that her devoted service had been highly appreciated by the committee, and that her skilful and sympathetic attention would be gratefully remembered by the numerous patients who had been under her care. The chairman, in making the presentation, referred to her approaching marriage, and Miss Sculby expressed, in reply, her heartfelt thanks for all the kindness which she had received in Thetford.

### GARDEN PARTY AT MORETON-HAMPSTEAD.

A very successful garden party was held the other day in aid of the funds of the Moreton-Hampstead Cottage Hospital, the grounds at Hayne being kindly lent for the purpose by the Hon. W. F. D. Smith, M.P., who built and endowed the hospital in memory of his father, the late Mr. W. H. Smith. The day proved fine, in spite of a threatening moorland mist during the morning, and the fête was well supported by residents in the neighbouring villages—which benefit by the hospital—and by visitors to the district. Tennis and croquet tournaments were held, and bowls, archery, clock-golf, and clock-croquet were also provided for the amusement of the visitors. Afternoon tea was under the care of the medical and nursing staff, and was much appreciated. The Cragford Brass Band performed at intervals, and played for dancing from 6 to 8 p.m. It is hoped that quite a nice little sum will be added to the funds of the hospital.

### MENTAL NURSES AT PLAY.

Some amusing incidents took place at the Isle of Wight County Asylum Anniversary Sports and Fête, which was held at Whitecroft. The asylum band, attired in comic uniform dresses, were led twice round the lawn, closely followed by a procession of gaily-costumed nurses and attendants, and patients with decorated bicycles. At the hat-trimming competition for men the competitors were allowed five minutes in which to make ladies' straw hats look beautiful by the aid of coloured crimp paper and pins. At the end of that time nurses were invited to the platform to try on the hats, in order that they might be seen to more advantage and be more easily judged. After the various competitions, open to both sexes, were over, the prizes were distributed, and while a male attendant found himself the winner of a brooch or a pair of fancy hat-pins, a female nurse was the recipient of a tobacco pipe and pouch.

### DISTRICT NURSING IN THE BLACK COUNTRY.

At the adjourned annual meeting of the Bilston District Nursing Association the question had to be decided whether the organisation should be continued on a permanent basis. Five years ago a small number of friends guaranteed the expenses of the movement for that period, and it had now to be determined whether the Association could stand on its own legs. The chairman was able to report that an appeal which had been issued for subscriptions had been well responded to, and he said that, with the aid of the contributions from workmen, subscriptions yielding £80 or £90 might be anticipated. A recent garden party had realised a net profit of over £25. In these circumstances, Dr. Ridley Bailey, who declared that it would be little short of a calamity if the work of the Association had to cease, proposed that it should be proceeded with, and the motion having been agreed to, a general committee was appointed.

### SUICIDE OF A HOSPITAL NURSE.

An inquiry was held last week at the Town Hall Brighton, relative to the death of a hospital nurse named Gertrude Laura Leeks, aged 38, who was found dead in her room. The evidence showed that death was due to suffocation combined with strangulation, the suffocation having been caused by a pair of cloth gloves pushed into the mouth, and the strangulation by means of a cord fastened so tightly round the neck of the deceased that the medical man who was called in had to cut it. He had no doubt that this had been done by herself. Letters written by the deceased, which had been opened by the deputy-coroner, indicated that she had been greatly disappointed in not obtaining appointments for which she had applied, and the jury returned a verdict of "Suicide while temporarily of unsound mind."

### VILLAGE NURSES IN THE EAST RIDING.

In the 13th annual report of the Wilton Beacon Benefit Nursing Association, which has just been issued, it is stated that the Society has now a staff of 21 nurses. The income last year was £662, and the balance in hand £48. From a financial point of view this is an improvement on the previous year, but the report says that the nursing staff is still not large enough to meet all the demands made upon it

### SHORT ITEMS.

Queen Victoria's Jubilee Institute for Nurses, Cardiff and District Branch, 14 Park Grove, Cardiff, has started a Maternity Branch at 12 St. Andrew's Crescent, Cardiff.—Kent and Canterbury Institute for Trained Nurses, 62 Burgate, Canterbury, has closed its branch at Herne Bay. The Home was called "St. Barnabas Canterbury Nursing Home." It was opened in 1901.—Bath Trained Nurses' Institute and Home for District Nurses are now divided as follows: Bath District Nursing Institute and Bath Trained Nurses' Home and Institute.

### The Nursing Outlook.

"From magnanimity, all fear above;
From nobler recompense, above applause,
Which owes to man's short outlook all its charm."

### AN ALL-ROUND TRAINING.

It is a misfortune to nurses and the public that the nurse-training in this country provides no adequate means whereby a probationer may acquire a thorough knowledge of private nursing. We have pointed out from time to time the discomfort to the patient and the unpopularity for the nurse which arise from this absence of definite training in the care of private cases. Where a probationer belongs to a great nurse-training school she is apt to become a mere item in a great machine, which is so constantly in motion that the pupil nurse stands in danger of losing such powers of initiative as she may possess. Necessarily the organisation of a great hospital has to run smoothly, and the whole of the workers are called upon to largely sink their individuality in subordination to this idea. The Scylla and Charybdis of the nurse-training school are in practice on the one hand the imperative necessity of systematic instruction in a large hospital, and on the other the danger of the probationer being so tied by routine and rush as to become mechanical in her movements and dependent in all things. No doubt some of the best educated and intelligent probationers under any system would rise superior to their surroundings and acquire a sound, practical knowledge of nursing in all its branches. Still the system is faulty and ill-suited in many respects to the majority of probationers, and new and better methods are called for in the interests of all concerned. It is a sad fact that at the present time no single hospital, however large, is so organised as to enable a probationer to acquire within its walls or under its ægis a thorough training in every branch of nursing. There is no reason why this state of things should continue, providing those in authority will set themselves to bring about a happier state of affairs. It is a great injury to many innocent workers in the nursing world that they should, from imperfect knowledge, go for training to a fever or children's or other special hospital, and should remain at work there for some three years under the impression that they are being qualified to follow their profession with efficiency at the end of such period.

General nursing is one thing; nursing confined to a special class of cases, quite another. We hope that before the present year ends a nursing conference may assemble which will be representative of the nurse-training schools and of each type of special hospital where nurses receive a portion of their training, for the purpose of securing a definite standard of efficiency which it will be possible for every probationer readily to attain through the co-operation of the authorities of these various institutions. We have frequently advocated, for instance, co-operation between the general hospitals and the lunatic asylums, whereby the nurses at the former would have the opportunity of being trained in mental nursing, whilst the attendants at the latter would receive a sound general training in nursing at the general hospital. All that is required is to secure the co-operation of the managers of the large and small hospitals and kindred institutions in each centre of the population for the purpose of providing adequate facilities for utilising the whole of the material at their disposal for the efficient training of probationers in every department of nursing.

In the United States an attempt is shortly to be made to establish a college or colleges for nurses in connection with the University. Each college is to have a four years' course devoted to the education of trained nurses. The scheme differs from those employed in hospital training schools, in the circumstance that it provides for the education of the nurse preliminary to the actual technical instruction in nursing. It is further contemplated that, in addition to instruction by lectures and study, all the graduates shall receive training experience in private homes with private patients, as well as in hospital wards. The scheme involves proposals somewhat similar to those we have ventured to make in this article, and aims at giving every graduate a thorough grounding in all branches of nursing prior to the issue of her diploma and certificates. No doubt the majority of the women who join one of these university schemes will belong to a class from which matrons and superintendents will ultimately be selected. There is a great want in this country at the present time of a system of higher education for nurses, as well as for the organisation of a system whereby the whole of the material available for teaching may be utilised with results which cannot fail to add immeasurably to the value which the medical profession and the public will then rightly attach to the calling of a nurse. The policy of isolated training, confined to a particular nurse-training school which keeps itself aloof from all other classes and fails to avail itself of all the material which its probationers ought to have placed at their disposal, is really responsible for most of the errors, as well as for the general deadlock at which nursing matters have arrived in this country to-day. Fortunately this view is now accepted by the majority at any rate of those who are immediately responsible for the training of nurses, and we certainly hope that another winter will not be allowed to pass without the assembling of a conference and the organisation of a thorough system of co-operation, whereby every facility will be afforded to the probationer of the future to become mistress of all branches of nursing knowledge, and so to claim that she is in fact, what she ought to be in reality, a thoroughly-trained and efficient nurse.

### Medical Electricity and Light Treatment.

By KATE NEALE, Sister-in-Charge of the Actino-Therapeutic Department, Guy's Hospital.

### V.—STATIC ELECTRICITY.

(*continued from p.* 326).

### I.—Simple Charge.

1. *Positive.*—In this method the patient is charged with positive electricity as he sits on the stool, and is allowed to remain quietly in that condition from ten to twenty minutes, according to the doctor's instructions. He feels no electric shock, but experiences a gentle and curious sensation over his whole body, as though threads of gossamer were twined about him. The hair on the scalp and face bristles, and a movement is produced of the finer down covering the trunk and limbs, and it is to this latter that the gossamer sensation is due.

To apply the treatment, seat the patient comfortably in a chair on the insulating stool and start the machine. Ascertain the polarity of the conductors by the method already given, and join the positive to the patient either by the crook, or, if the stool be provided with a brass plate, by a chain, one end of which must be twisted round the conductor, and the other attached to the plate. Earth the negative conductor by another chain passing to the hook on the glass case. Continue the treatment for the prescribed time, and then stop the machine. Discharge the patient by placing one foot on his stool, or by allowing him to remain for a few minutes until his charge has escaped into the air.

2. *Negative.*—The procedure is exactly the same as for positive charging, except that the patient is connected with the negative conductor, and the positive is earthed.

### II.—Charge and Discharge.

1. *Positive.*—This method differs from the preceding, in that the patient is alternately charged and discharged throughout the whole time of treatment. Connections are made as before, but in addition a ball electrode, properly earthed, is placed near the positive conductor, so that a stream of sparks passes from one to the other. It is best to use a special stand to support the electrode; if you hold it yourself, the sparks will vary in size and frequency, owing to the difficulty of keeping absolutely still. The principle of the treatment is simple. The patient becomes charged with positive electricity until a spark passes from conductor to ball electrode, when he becomes discharged. He again becomes charged, and again the passage of a spark redischarges him. With each charge and discharge the hair on the head can be seen to rise and fall. Many patients find the incessant crackling of the sparks intolerable, and the treatment is therefore not often used.

2. *Negative.*—Apply the same treatment as in the previous case but substitute the negative conductor for the positive.

### III.—Static Breeze.

1. *Positive.*—I have already alluded to the breeze a patient feels from neighbouring objects, and the same effect is produced when an earthed electrode is brought near. In treating with the static breeze, you direct at the patient, from a distance of about six inches, either a pointed or forked electrode, earthed by a chain. With this interval a slight effect may be felt if the machine is working well, but to obtain a breeze of the required strength you must approach the electrode nearer, perhaps to a distance of three inches or so, though you must be careful not to bring it close enough to provoke a spark. Played upon the bare skin the breeze produces a refreshing and cooling sensation, but directed through the clothes a hot, prickling feeling is the result. When the doctor has ordered the application to one special region of the body you must point the electrode steadily at the area, but no patient will endure this for long owing to the burning feeling produced. More frequently a moving breeze is required, and this is applied to, for example, the spine, by directing the breeze up and down the back with much the same movement you would use in watering with a hose. The term **positive breeze** is applied to the treatment when the patient's charge is negative and the breeze from the electrode is positive. And vice versa to apply

2. *Negative Breeze.*—The patient is connected by the crook to the positive conductor. In all cases remember that a strong breeze, unexpectedly delivered, is unpleasant, and you should warn the patient when about to change the site of application. With a negative breeze the electrode must be held further away from the body to avoid risk of sparking.

3. *Head Breeze.*—So many painful conditions of the head are relieved by the static breeze that a special arrangement has been devised to overcome the difficulty a person of ordinary stature finds in applying treatment to the crown of the head. As shown in fig. 7, a tassel electrode hangs from an earthed rod attached to the top of the machine case. The rod can be moved in various directions and allows the adjustment of the tassel at a suitable height above the patient's head. As soon as the breeze begins to play the hair usually stands on end.

### IV.—Static Sparks.

1. *Positive.* 2. *Negative.*—The most vigorous application of static electricity is obtained by approaching the electrode so close to the patient that an exchange of sparks is made. The treatment is not always pleasant, and may in careless hands produce serious injury. Never bring an electrode near enough to the face or any sensitive part of the body to produce a spark, and never treat a knee or elbow or any surface which is protected only by a thin covering of soft tissue with any but short mild sparks.

Charge the patient with positive or negative electricity, according to whether the sparking is to be negative or positive and treat with a ball electrode, earthed as usual. Your object is not to deliver a rapid shower of sparks but to administer the treatment spark by spark. So long as the ball is within sparking distance, a crackling volley will pass, but by rapidly approaching the electrode to the patient and at once moving it away ag[illegible]in, time will be given for the exchange of only one spark. To accomplish this manœuvre requires a little practice. You know how you shake down a thermometer. Execute just the same motion with the electrode. In this way the ball will move in the air along a curve, and for only a fraction of a second will it be near the skin. At that moment the spark will fly and the electrode will have passed on its curve before another can leap across. If the action of the ball electrode is too forcible, substitute the point electrode, or better still, weaken the sparks by placing one foot on the insulating stool, thus diminishing the charge in the patient.

In sparking, even more than in breezing, you must be particular to warn the patient when and where you are about to administer treatment. Women are usually somewhat timid of the sparks, but small children often regard them with genuine fear, and on th[illegible]are occasions it is prescribed for them you [illegible] need to exercise all your persuasive powers before being allowed to proceed.

3. *Roller.*—The patient is charged as usual, positively or negatively, and treatment is applied through the clothes by means of an earthed roller. As this is moved up and down lavish showers of sparks pass to the patient producing a painful tingling sensation followed by an after feeling of heat. The thicker the clothes through which the sparks are given the severer is the effect. Therefore remove one or more garments to moderate the treatment; to intensify it wrap in a rug. The roller must be moved rapidly and used only for a few seconds at a time.

**Dangers of Treatment.**

The dangers are chiefly those which arise in connection with accidental sparking. A spark near the eye or on the face may result in serious harm and the same may occur in other and sensitive parts of the body such as the neighbourhood of the heart. Remember the warning I have given you to allow no person or thing to be close to the patient when charged, and do not forget that so long as you are holding the electrode near the patient you have in your hand an implement capable of inflicting injury. Take care then how you move it about lest a spark flash out unintended. If the above precautions are observed neither simple charging nor breezing has any special attendant danger, but sparking itself, and still more, roller friction, often bring in their train redness and burning sensations of the skin and may even go so far as to produce definite weals on the body.

**Diseases Influenced by Static Electricity.**

Static Electricity has a peculiarly invigorating effect on the system as a whole, and medical men appear to be agreed that as a local application it quiets many painful conditions. The cases you will be instructed to treat will comprise examples of sciatica, neuralgia, and headache, in all which the static breeze often produces marked improvement. Insomnia is another condition frequently relieved. Electric sparking is of course, more stimulating in its effects and therefore you will find it enjoined in such a condition as paralysis of muscles.

### The Nurses' Clinic.

### THE CHILDREN'S WARD IN HOT WEATHER. BY A MATRON.

Nurses are more often alive to the necessity of keeping their little charges warm in cold weather than of seeing that they are not over-heated in the summer time, and it is certain that simply through want of thought many little ones in our children's wards suffer much unnecessary discomfort through this very thing.

Who has not seen a tiny infant smothered in blankets on a sultry day, its curtains drawn and hardly an aperture left to breathe through, because the nurse, who is accustomed to wrapping the babies up warmly during most of the year, is oblivious to the fact that the temperature of the ward is nearly or quite 80°. Or again, a blazing midsmmer sun at its noonday height may be shining full into the face of another child, too ill or too young to voice its complaint other than by turning its head restlessly from side to side or crying fretfully.

A great deal of whining and tears, besides real pain and needless misery can be averted if the nurse in charge exercises a little thoughtful care in the matter. Blinds can be lowered before the window-panes become as burning glasses for heat, and raised again as soon as the sun is off that side of the ward. A fretful child, taken out of its cot for a few minutes while its mattress is turned, will, if sponged with cool water and given a few spoonfuls of cold water—of course boiled—often go to sleep when put back into a cool bed, and lightly covered with a sheet or one thin blanket.

At night, when the little ones simply will not keep covered, and yet, when the early dawn is apt to be chilly, a good plan is to cover the lower part of the whole cot with a large blanket, which, hanging right over the cot-rails, does not touch the child at all or impede his movements, and yet keeps off draught.

Another point apt to be overlooked in the management of children is the increased thirst caused by the hot weather; this need is extremely likely to be met by kindhearted, yet unreflecting nurses, by constantly giving them milk to drink, which is a great mistake. Milk is a food, not a drink only. It cannot quench thirst like water, and often sets up indigestion by overloading the stomach. If given at all for the purpose of quenching thirst, it should be largely diluted with cold boiled water or barley water. The latter, alone, or with a squeeze of fresh lemon in it is a most wholesome summer drink for children, and one generally taken readily by them. The old-fashioned toast water is another harmless, thirst-quenching and slightly nourishing drink, while home-made lemonade is excellent.

After operation, too, a child is apt to be overheated in summer by its well-meaning attendants. As soon as it is returned to bed, it is immediately covered with one or two blankets tucked tightly round it, and fenced in on every side by hot-water bottles, as the nurse's standing orders are so to treat every case after operation.

This, as a general rule, is perfectly right treatment; but in summer, is a rule that is best interpreted by individual common sense.

If we consider a moment, we shall remember that the great point is to keep the patient warm and so minimise shock as much as possible, and though we all know that a badly collapsed patient will be cold and clammy to the touch even on the hottest day, still if he is nothing of the

### THE NURSES' CLINIC—*Continued.*

kind after the anæsthetic, why add to his sufferings by unnecessary blankets and hot bottles? One light blanket and a hot bottle, removed as soon as the child begins to "come round" are all that are required, and will conduce to restfulness. "Feverishness," says Florence Nightingale, "is generally supposed to be a symptom of fever—in nine cases out of ten it is a symptom of bedding."

Common sense, not a blind following of stereotyped rules, is greatly needed even in these days of modern nursing. Plenty of open windows, cool bathing, light clothing, simple summer drinks (not iced unless by the doctor's orders) should be a nurse's aim to provide for her little ones in a hospital ward in summer time. If there are outside blinds, balconies attached to the wards, or good grounds belonging to the hospital, it is of course much easier to procure the coolest and freshest air possible. Cots can be wheeled on to the balcony and kept there all day long, except when the sun is too hot. If there is a garden, the children can be carried out and laid on old mattresses on the ground, so that there is no danger, as when placing them in bath chairs, etc., of their falling and hurting themselves. These mattresses, if covered with mackintosh stitched to both sides and with pillows also covered with mackintosh, entirely obviate any danger from damp. White linen hats and sun-bonnets, or very light straw hats are necessary, and the mattresses require moving from time to time so that the little ones are not exposed to the full glare of too bright a light in their eyes. Thick coats or dressing-gowns, heavy rugs and blankets are not needed, and the less covering they have in the very hot weather the better.

There is no doubt but that the nursing of children in summer is trying work for both children and nurses and calls for much patience and forbearance, but no really good nurse will grudge the extra trouble and time which it takes to make the little ones as comfortable as possible under conditions which give every excuse for added languor and fretfulness.

### With the Lourdes Pilgrims.

BY AN ENGLISH NURSE.

The "White Train" started at 4 p.m. on August 17th. Early in the day great vans of mattresses, pillows, food, and all manner of necessaries had been brought from the Convent of the Nursing Sisters. These nuns undertake the actual nursing of the sick pilgrims on the journey to and from Lourdes. Fourteen pilgrim trains, in all, leave Paris on that day for Lourdes, and each is known by the colour of the flag it carries. About 300 of the worst cases, and about 500 other people—mainly nursing nuns, lady hospitallers, gentlemen "brancardiers" (bearers), priests, and friends of the patients—travel in the "White Train."

### Arrival of the Train.

We reached the Gare d'Orléans at about 3 p.m. Mother Brigitte, I, and Mary (an Irish girl crippled with chronic rheumatism), came in a fiacre together. Mary's hands were deformed and almost useless, and she could only hop slowly on crutches. She had spent months at a time in hospitals, and was declared incurable. In her pocket was the "Manual of Devotions," and the letter (both in French!) that every "hospitalised" pilgrim receives from the Paris Committee; and round her neck, on a string, was a card bearing her name and number. We had great work to get her through the struggling crowd at the entrance to the station. Once inside, all was well. "Brancardiers," with stretchers and invalid chairs for those who need them, are on the look out for the pilgrims. One looked at Mary's card; two lifted her into a chair and carried her to the door of a compartment marked with an immense white "7."

### Hospital Ward in Third-class Carriage.

Five compartments of a third-class carriage represent a ward! The great white figures distinguish these divisions, and a card with the names of a lady and two nuns in charge of that division, is on the door. The nuns wear white nurses' aprons and sleeves, and on the apron bib is a large red cross. They have the cheerfully efficient air of nurses accustomed to meet and cope with emergencies. Mother Brigitte and I looked into her ward. We could see over four compartments, but the end one was separate. The nun took note of all, saw that the great water-can, with a long-handled ladle attached, was filled, that a broom, utensils and brushes, and bowls were under the seats. Then the patients were arranged with as much room for each as could be spared, or was absolutely necessary. All night they would be in the train. The next day and night they would rest at Poictiers. All the day and night after that they would travel again. From the van that had been converted into a "dèpense" our special brancardier brought us bottles of milk, bouillon, and wine. Baskets and packages of food brought by the patients, were stowed away under the seats. Hats, wraps, and baskets were hung up on the hooks.

### Some of the Patients.

Imagine 300 patients—some actually dying, many dangerously ill, many quite helpless—to be attended to for days and nights in the narrow third-class carriages of a train, without corridor or lavatories! Mother Brigitte sat against the window, whence she could see all the four compartments. I sat beside her. A woman lying on a mattress took up all the rest of our side. Opposite us sat three women who would lie down on the two mattresses as they must or could. Mary, at full length, and several other patients, were in the next compartment. The other nun was in the fourth compartment, and the lady in the third. Only those patients who must lie down always, or generally, had room to lie at length. Nuns and ladies always sat up. A terribly deformed little hunchback was in the fifth compartment, and his aunt was with him. No one there was too ill to be left between the times when the train stopped. The train started to the sound of the "Magnificat" sung by the passengers. In spite of open windows and green blinds the heat was terrific, and the smell from disease, unwashed humanity, and the parcels of food added to the discomfort. The patients seemed tired and unenthusiastic. They joined languidly in the prayers and hymns that were started at regular intervals throughout the train.

### Neither Comforts nor Conveniences.

At each stop nuns, ladies, and brancardiers helped the patients to get out of the train for change of air, more room, or any other need. Sometimes there was a long stop and no station; sometimes a short stop at a station; and the scenes on either occasion were startling to English eyes. Luckily there were no English except one or two nuns, and I, a nurse. When our patients were settled we strolled up and down, and chatted with our friends. In number 8 Sister Lucie sat fanning a woman dying of consumption. At one station I was seized and embraced by a big, handsome American nun. She and a friend had read a paper of mine in The Hospital about the nursing nuns, and entered the New York Convent. She blessed me as her benefactor. Before each start the nuns see that every one is in his place, and there is often a frantic rush of brancardiers, ladies, and nuns to find and bring in stragglers. Fresh water, milk, and bouillon are brought as they are required, and neatly packed baskets of provisions arrive, at fixed times, for the nuns, and any patients unprovided for.

### A Wearisome Night.

During the night silence was enforced, but several of the patients required frequent attention. Mattresses kept slipping, pillows went wrong, and changes of position were necessary for the tired, uncomfortable patients. Between times Mother Brigitte and I sat bolt upright in our narrow space between the window and the woman's feet. After midnight all who possibly could fasted in order to go to communion in the morning. We reached Lourdes at dawn on the 20th. The sick were conveyed to their hospitals, where the men are nursed by gentlemen and the women by ladies, day and night. The beds touch each other, and clothing and parcels make apparent disorder. But rest, dressing of wounds, feeding, and visits to baths, grotto, and processions are as punctual as hospital routine. Outside wait the gay, devoted brancardiers I saw some of them peeling potatoes in aprons and gloves.

### Waiting to be Cured.

The patients are bathed every day, waiting their turn in an enclosed space. In the afternoon they are ranged in long rows, on stretchers, chairs, and benches, on either side of the way left clear for the Procession of the Blessed Sacrament. Between times they rest before the grotto. Everywhere great crowds, led by priests, are praying for the miracles of healing. The air vibrates with prayer. The dying patients who were bathed lingered on. One died on the third day at Lourdes; another on the way home. Both knew the pilgrimage meant a miracle or death. Many, who must die, hope to die at Lourdes. Mary walked up and down stairs without crutches before she left, and has been better ever since. Two babies with sloughing sores on their legs came out of the bath with dry scars! These particular facts came under my notice, and I simply record them. During the procession on the third day five times a roar from thirty thousand pilgrims announced a miracle. But I was never near enough to see what happened. Improvement in many cases, calmness of mind and contentment in nearly all, and a great longing to come back to Lourdes next year, were what struck me most in the patients on the return journey.

### Incidents in a Nurse's Life.

**Contributions for this column are invited.**

### HEARTS OF STONE.

*Monday*, 10 a.m. — On this particular morning two women were sitting in the out-patient department of The General when a nurse passed through carrying in her arms a child of three or four years wrapt in a blanket. The child's face was just visible; it was very white and still. The nurse walked quickly, buoyantly. She was evidently happy.

"Hullo, nurse!" I exclaimed, "have they consented to Boy Blue's operation?"

"Yes; isn't it good?" she answered as she passed me.

"'Ear that," one woman said indignantly to the other giving her a nudge. "Really, these nurses 'ave hearts of stone, I do declare. That poor little chap's a-going to 'ave a operation and they're as pleased as can be. I believe they just love to see 'em sliced up, same as the doctors do. They ain't women, they ain't."

. . . . . . .

10 p.m.—"Come in."

In answer I opened nurse Elizabeth's bedroom door expecting a pleasant half-hour's chat, but my hopes fell when I saw her sitting on the floor in the middle of her room. Her shoes had evidently been kicked off; they lay on either side of her in a forlorn manner. Her collar was reclining in the far corner, cuffs about to follow, and tears were running down each cheek without any attempt being made to stop their progress. I sat on the bed the better to survey this unusual spectacle.

"What do you want here?" Elizabeth growled.

"Well, my dear Queen Beth, first tell me what ails you? Favourite courtiers contemplating speedy marriage, or sister suffering from a spell of nerves?"

No answer.

I put my head on one side and murmured,

"The probationers that come in the spring Tra la!
Have something to do with the case."

A pause. Elizabeth then began to attack the unfortunate hairpins in her fiery mane which she proceeded to shake over her shoulders.

I tried again. "Really, Queen Beth, I have often seen you tidier but never more beautiful than now."

This had some effect.

"Get away, Nurse, I want to be alone, I detest hospital, I detest nursing, I detest op's. I wish all Pro's were at the bottom of the sea; I'll go home to-morrow, 'pon my word I will."

"So this is the dignified end of a week's staff duty," I commented.

"Please say what you want and go," Elizabeth answered severely.

I walked to the door remarking,

"And that's what I mean when I sing or I say
Oh bother Probationers that come in the May."

"It's all very well," she hurled the words at me, "for you to stand there and sneer, you'll soon be in my shoes your self and then——" A sudden movement warned me. "Is it sixes or sevens you take," I queried under my breath and closed the door hastily. There was an ominous thud! Elizabeth must take eights!! And her heart is surely made of fire and brimstone.

*Following Morning*, 6 a.m.—Nurse Elizabeth always needs a second call and a cup of tea at 6 a.m. So I intended when I took it to inquire what time she was going to start home. What was my astonishment then to find her this morning up and radiant, humming to herself as she changed her cycling costume for uniform!

"Been out?" I gasped.

"Ah! Nurse, isn't it good news? Boy Blue's doing well after all, Night Sister says Mr. Russell gives every hope."

"What's that to do with extra early rising and cycling?"

"Well, you silly! can't you see? Sister gave me permission to cycle over to Canbury and tell his mother. There's no carrier until Saturday you know, and she was so dreadfully anxious poor little woman!

. . . . . . . . .

Surely, after all, Elizabeth's heart must be made of flesh and blood.

### To Nurses.

**We invite contributions from any of our readers, and shall be glad to pay for "Notes on News from the Nursing World," or for articles describing nursing experiences at home or abroad dealing with any nursing question from an original point of view, according to length. The minimum payment is 5s. Contributions on topical subjects are specially welcome. Notices of appointments, letters, entertainments, presentations, and deaths are not paid for, but we are always glad to receive them. All rejected manuscripts are returned in due course, and all payments for manuscripts used are made as early as possible after the beginning of each quarter.**

### The Naval and Military Hospital of Spezia.

BY AN ENGLISH MATRON.

During a recent visit to Spezia I had the good fortune of being permitted to visit and fully inspect its large and important Naval and Military Hospital. Spezia is the first military port of Italy, also an important naval station, and possesses an arsenal of European renown, which gives employment to over three thousand workmen. The hospital is situated at the western end of the city, and is surrounded by extensive gardens dotted with shady trees, and decorated with sweet-scented flowers. It was built about the year 1870, and contains beds for three hundred patients. These include soldiers and sailors, officers of both services, and any cases of accidents which occur at the arsenal. There are twelve wards, each containing twenty-two beds, besides special wards for infectious diseases. The wards are light and airy, with red tiled floors, and white walls and ceilings. The beds are of iron, covered with white quilts. Beside each bed is a marble shelf fixed into the wall, upon which stands a shining bottle and glass, and other conveniences. At the back of each is a black slate, fixed also to the wall, bearing the name and disease of the patient, date of admission, and class of diet. Two or three marble-topped tables are scattered about the ward, on which is served the patients' food, and at which the convalescents assemble at meal hours. The meals are served as follows: coffee and bread at 7 A.M., soup and meat at 10.30, and a substantial meal again at 3.30. There are five classes of diet, regulated according to the patients' requirements.

### The Staff.

At the head of each ward is a "Suora," or Sister of Charity belonging to the order of St. Vincent de Paul. Under her are two orderlies, who appear to do the actual nursing. There are sixteen sisters in all, and about sixty orderlies of three different grades. The Director of the hospital, who is a medical colonel, lives in a pretty house in the hospital grounds. Under him are a number of medical officers, varying in rank from majors to sub-lieutenants. There are always two medical officers on duty for 24 hours at a stretch. They wear uniform when on duty, also the long white jacket usual on the Continent. The rank and file of the army and navy are admitted gratis, but all officers pay according to their official grade. There is a handsome pavilion recently erected for the benefit of the officers, fitted up with the latest improvements and conveniences.

### A Study in White.

Separate rooms, handsomely furnished, are provided for each officer, also dining-rooms and a library for convalescents. In this pavilion is situated the operation-room, which is a perfect "study in white." It is lighted by four large windows and a sky-light directly over the operation-table, which is made of white enamelled zinc, revolves easily, and is fitted with receptacles beneath for disinfectants. The floor is of white Carrara marble and the walls, to a height of over six feet, are of the same material; all corners are rounded to allow of thorough disinfection. Four different sets of hot-water pipes are provided, so that the heat of the room may be raised to the required temperature. The doors are of opaque glass, as are also the doors of the adjoining rooms devoted to the care of instruments, surgical appliances, and sterilising apparatus. The dressings, after being sterilised, are placed in large copper receptacles. Powerful light by electricity permits of operations being performed at night with as much facility as by daylight.

### Wards and Patients.

The entire building is warmed by hot-water pipes and is ventilated artificially. One special ward is set apart for a receiving-room, where the patients remain under observation for a few days; another is used as a surgery for first aid, and the sunniest of all is reserved for tubercular patients. The staircases are all of marble, with light painted walls. No smoking is allowed in the wards, but the convalescents may smoke in the gardens *ad libitum*. The patients all wear white caps of the pattern familiarly called night-caps in England, ending off in a point with a white tassel. Readers of Dickens will remember the style worn by the immortal Scrooge. They also wear blue suits in cotton or cloth, according to the season, when out of bed.

### Hard-working Sisters.

In the linen-room I saw the Sister Superior who is a stout buxom lady, none the worse for long years of early rising and incessant work. The Sisters all wear the uniform of their order—namely, white floppy caps, blue cloth dresses with open flowing sleeves and ample white aprons. They rise at four in the morning and retire at nine at night. They have no holidays and their chief recreation is going to Mass.

### The Laboratory and Laundry.

The dispensary is a busy place, presided over by the chief dispenser, who is aided by a staff of assistants all wearing blue cotton jackets. I visited the laboratory where a great deal of work is done by the bacteriologist. Here, the doctor who was acting as my guide, allowed me to see through a microscope some tubercle bacilli, it was difficult to believe that those small spiral germs could work such havoc in the human body. I also saw some small scales destined to be used in weighing very delicate matters, the aforesaid scales having cost a fabulous price. Any local doctor may send specimens of sputa, etc., to be examined at this laboratory for the small sum of five francs = four shillings. The hospital is supplied abundantly with good drinking water, and gas is always ready in case the electric light should fail at any time. The large steam laundry is in charge of two Sisters, and they cheerfully assured me that they were quite happy in their department, although it seemed to me it must mean wet feet, a vapoury atmosphere, and that indefinable smell which accompanies the process of perpetual washing. An inner room revealed a number of women engaged in repairing linen, and a conspicuous object was a Singer's sewing-machine; this probably was worked by the Sisters.

### Sailors as Nurses.

In the gardens were some rooms occupied by insane patients and also rooms used as sleeping apartments for the sailors acting as nurses. There is also a local fire brigade, which is attached to the hospital. Detached from the main building is a mortuary, also a dissecting-room and disinfecting chamber. The library for the use of the staff is stocked with the latest scientific works.

### Adepts in the Art of Cooking.

The culinary department is, like the laundry, superintended by two Sisters, who seemed to be adepts in the art of cooking, judging from the savoury broth, appetising omelettes, and cooked meat which I saw distributed to the patients. Of course macaroni in some form is an item of the daily diet, also the light wine of the country. By means of a "lift" all the food is conveyed from the kitchens to the upper stories of the building. The general cheerfulness of both patients and staff was very striking, and seemed to show entire satisfaction with the arrangements for the comfort of all alike.

### Treatment by Electricity.

The most wonderful sight of all is the room for treatment by electricity. This was exhibited with honest pride by the young sailor who evidently had charge of this department. Being a sufferer from neuralgia I was permitted to experience electric shocks, both of the "interrupted" and "continuous" order. Then an electric hot-air bath was lighted, and the sailor seated himself within to show me how it worked. Then I was invited to seat myself in what looked like a dentist's chair, and having removed my nurse's bonnet, an electric current was turned on which played through my hair in the most delightful way. Subsequently, the doctor took his seat in the chair, and I had the pleasure of seeing his hair literally rise from his head. This I was told was excellent treatment for neuralgia of the head. I was next shown an electric bath which had perforated holes all round it for the passage of electric currents; this looked rather alarming. Then came a large cage in which the patient stood upright and was entirely surrounded by powerful electric currents. The blinds being lowered, I saw the working of a magnificent Röntgen-ray apparatus. I must not omit to mention the presence of a Finsen-light appliance and a dark room for developing photographs. I was presented with the photograph of a human hand as seen through Röntgen rays. I began to wonder if this military hospital lacked anything which could be used for the benefit of suffering soldiers and sailors. Certainly it seemed to be thoroughly efficient in every department.

A Parting Gift.

My guide accompanied me to the gate of the hospital, and there ordered the gardener to pick me a charming bouquet of flowers, while he showed me a small room where the medical staff keep their uniforms when off duty.

I am greatly indebted to Maggiore Dottore Giusti for his kindness in giving up so much time to show me everything of interest in the hospital, and to Signore Dottore Ballerini for having given me a letter of introduction which procured me the pleasure of such an interesting visit. The latter is a highly-esteemed medical officer of health for the city of Spezia.

### A Day in a London Infirmary.

BY A NURSE.

Clang! clang!—and we wake from blissful dreams of home to the fact that it is 6 a.m., and our day has begun. Then there is a general stampede to the bathrooms, the first "tubbers" being earnestly exhorted to hurry up.

Clang! clang! again—this time it is for breakfast, and downstairs we rush, for punctuality is a cardinal virtue here and laggards have to confess their sins of tardiness with much humility.

By 7 a.m. we are in the wards and our day's work has started in real earnest. Helpless patients have to be washed, beds made, wards swept and dusted, lockers polished, flowers arranged, with of course the thousand and one interruptions that are bound to occur. However, by 9 o'clock everything is in order, and we are not sorry to adjourn to the trim little ward kitchen for lunch. This morning it must be a very hurried meal, for there is an operation at 10 a.m., and the theatre has to be prepared. We have no theatre sister nor nurse, and each ward is responsible for its own operations. With a little extra hurrying up it is all ready and the appointed hour finds us clad in sterilised pinafores with bare, well scrubbed arms waiting for the surgeons. The patient is a burly gentleman who takes a lot of putting to sleep, and at the start much force is required to hold him down, but at last he subsides and the performance begins. When the final bandage is adjusted, we cannot help giving subdued sighs of relief, and we assist the operators to doff their gowns and don their coats with great alacrity. Before the victim is fairly settled in bed the junior resident arrives on the scene, and has to be escorted round. While he is still on the war path, with a creak and a groan up comes the lift, and the receiving wardsman hands over two very decrepit new patients—an aged "daddy" of 88 with bronchitis, and a bad heart case. Then comes the great event of the day, so far as the patients are concerned—viz., dinner. Helpless folk have to be propped up with bedrests and pillows, and lockers cleared, for the lift is groaning its way up again, this time with an appetising load of food. By the time it is all carved and distributed the clock points to our dinner hour, and we are not sorry to descend and refresh ourselves. Three-quarters-of-an-hour is the allotted time, so we can spare twenty minutes or so for a stroll in the big, shady garden, a most delightful spot, with its old trees, flowers, and green lawns. On our return the wards do not present a picturesque appearance, but in a short time everything is in order, and then follows the quiet hour for the patients, while we retire to sponge the high temperature patients (enterics, pneumonias, &c.), and then make preparations for tea; also there are big piles of fresh linen waiting to be sorted and packed away in the capacious cupboards. Four p.m. is tea time, and we hurry round with piles of bread-and-butter, steaming jugs of tea, and eggs for the favoured, our small boys getting bread-and-jam. Our own tea half-hour follows, and then the evening's work begins in full force. We are just struggling through with the last beds, and devoutly hoping to be able to spare time to make up theatre dressings, when the receiving wardsman appears once more with the not too welcome tidings that there is a stretcher case on its way up, a bad pneumonia, and almost before we have time to get the sponging blankets in the patient arrives. Strychnine and brandy are the first orders, and before it is time for lights down we leave him comparatively comfortably installed in a steam tent with a kettle bubbling. Such a number of odd jobs have to be tucked into the last half-hour, but just as the night owls appear we finish up and join the happy throng who are making for the supper-room. Then follows another stroll in the garden (if it is not wet), and by 10 p.m. we are quite ready for tubbing and bed, feeling we have earned our night's repose.

### Novelties for Nurses.

(By Our Shopping Correspondent.)

### HANSEN'S JUNKET TABLETS.

It is doubtful whether the value of junket as a food for invalids and children is fully appreciated. Possibly its limited use is due to the usual method by which it is made. When a small quantity of rennet is to beused it is often regarded as wasteful to open a bottle of the essence which may not be used again for some time. Hansen's junket tablets remove every difficulty. Few dishes are so easily and quickly prepared as junket made with the use of these tablets. One tablet added to a quart of lukewarm milk is all that is necessary to secure an excellent junket, ready for consumption after the lapse of an hour. The price is 6d. for a tube containing 24 tablets, so that junket may be regarded as one of the most economical as well as wholesome forms of food for the invalid. Many persons will take junket readily who refuse milk in the natural form. The tablets can be obtained through grocers, or, if any difficulty arises, Mr. Sorensen, of 77 St. Thomas' Street, S.E., will supply them. Mr. Sorensen is prepared to provide the tablets on special terms to hospitals and institutions. In Australia and Canada they are largely used in medical institutions.

### Invalid Cookery of Old Times.

In the seventeenth century cooking for the sick must have been a much more elaborate affair than the simple ménu which we now think suitable for disordered digestions, if we may judge from the recipe-books which have come down to us. In the year 1601 Mr. John Shirley put forth a little brown volume, which he called "The Closet, or Treasury of Commodious Conceits and Hidden Secrets," containing, besides numerous every-day dishes, instructions for many of the homely remedies which it was the business of every good housewife to dispense to her household and dependents. From it we may learn how to prepare conserves of many kinds, from the conserve of roses, which "comforteth the stomach, and is good against the black coler," to conserve of bugloss "which is good for the frantic and the lunatic, and taketh away heart-burning." Perhaps what strikes the modern reader chiefly in the instructions is the quantity of food to be made. For instance, it is a simple thing nowadays to get a cup of chocolate ready; but these are Mr. John Shirley's directions for making it. "Take two ounces of chocolate, eight eggs, $\frac{1}{2}$ lb. sugar, white wine, rose-water, an ounce of mace or cinnamon, whichever the party fancies, and a gallon of milk. Boil them together over a gentle fire." We should imagine cock ale to be most likely a sort of chicken broth, but we may well be doubtful as to its suitability in an emergency, when we notice the time necessary for its preparation. The cook must take a young cock, and, having stoned four pounds of "raisins of the sun," must boil them and him in fair water. While this is in process she slices four netmegs, adds mace and a half a pound of dates, beats them well, and puts them, with two quarts of canary, into the broth, in which the cock is to be boiled to pieces. The liquor has then to be strained and put into a barrel, and after two months it will be fit to drink! Mr. John Shirley was a good patriot and also a bit of a vegetarian upon occasion. He thought that liquors made of English fruits, were most natural and healthful for English bodies, and that the fruits themselves much contribute, if advisedly eaten, to the increasing good blood and helping appetite and digestion. He specified the kinds of fruits that will benefit the invalid according to his disease. Roasted apples help melancholy and are good against pleurisy. Plums are good in hot diseases, pears cheer the heart, and nuts and philbers remove obstructions in the windpipe and fatten the body and breed little blood, as being of a dry constitution.

Very minute details are given for feeding a woman after child-birth. She must not be allowed to sleep for six hours. (This superstition still holds good in many country districts. Only this year a nurse was assured that her patient's sister would sit with her and keep her awake after an exceptionally bad confinement for a couple of hours, lest the poor woman should go off to sleep and never wake.) After the six hours she might be given a restorative of white wine, eggs, and St. John's wort for external use, and fed with caudles and nourishing liquids till the tenth day, when her temperate diet was to be changed for solid food. Of course there are many recipes for making caudle, but the chief and unvarying ingredient is ale, the strongest that can be got. It was not an exclusively feminine diet, as appears from the following directions for making it: "$1\frac{1}{2}$ pint of strong ale, 20 Jordan Almonds wiped clean but not blanched, 12 inches of the pith of young beef, two dates stamped small. Boil all together till it is a little thick, and give the party in the morning fasting six spoonfuls, and as much when he goeth to bed."

"The Accomplished Ladies' rich closet of rarities, or the Ingenious Gentlewoman's and Servant maid's Delightful Companion," condescends to provide four recipes for spoon meat, besides one for panada. A French barley posset, the newest fashion, requires two quarts of fresh milk and three pints of sweet cream, flavoured with mace and cinnamon, and finished off with a pint of white wine. An excellent chicken broth has a quart of Rhenish wine in it, and the fowls are stewed down with two ounces of China root, whatever that may be. Barley pottage and an "excellent white pot," which appears to be a sort of cabinet pudding, are the only other spoon meats described.

Both the little books describe a sovereign water, made by "Dr. Stevens, Physician," a man of great knowledge and cunning. "Therewith he did very many cures and kept it always secret, till of late, a little before his death, a special friend of his did get it in writing of him." The cure begins by taking a gallon of good Gascoyne wine, mixing in it a dram of each of many sorts of spices, and many handfuls of various herbs, then stirring it and distilling it in a limbeck. The first pint distilled is accounted the best, but a second pint of inferior quality might be extracted. This wonderful concoction had many virtues, curing everything from toothache to stone; and though it did not pretend to confer perpetual youth, yet "whoso taketh his water not too oft, it preserveth him in good liking, and maketh him seem young very long." The dose is small, one spoonful once a week! Dr. Stevens tested its virtues for himself, for we read that "it preserved him so that he lived eighty-eight years, whereof he lived ten bed-rid."

Another excellent recipe — but they are all excellent, according to the authors—is one to restore strength in them that are brought low with sickness. Take $\frac{1}{4}$ oz. of the brawne (flesh) of a pheasant and of a capon, steep them for two hours in rose-water, mixed with cucumber seeds, barley corns, spices, and a dozen other things, "seethe them all together and make lozenges thereof; whereof one is sufficient at once dissolved in a mess of pottage; this do, two or three times a day." One naturally wonders how much strength was restored by the lozenge, and how much by the pottage.

Among the more especially medicinal recipes is one for an excellent wine against the plague, pox, measles, smallpox, spotted fever, or any infectious disease. It is made by taking the best old Malaga and boiling it with treacle, maidenhair, balm, sage, and saffron. These herbs are to be gathered at particular times of year; and there are also instructions as to where they are to be picked, as the herbs growing in the country are better than those found in the towns, and there is more virtue in them when they grow upon hills, as they are not so fat, and grow smaller.

Of course there are many recipes for personal decoration. One elaborate wash will make a young face exceedingly beautiful, and an old one very tolerable! And another, compounded of sheep's milk and swallow's oil, makes wrinkles disappear.

Even barley water to our great grandmothers was not the very simple beverage that our sick cookery books teach us to make, though it is economical compared to the directions for nearly every other drink. The ingredients are a pennyworth of "liquorish," a pennyworth of raisins of the sun, a pennyworth of barley, and about two quarts of water. It is to be boiled down to half the quantity and strained, "and when it is cold, drink it with what appetite you may."

The pages of both our little books are redolent of country life. We can imagine the Lady Bountiful of the village busy in her still-room, superintending her maids in making hart's horn jelly for her sick folks. The inside of the horn was shaved finely, and boiled with two ale-quarts of water·

Then it was beaten with whites of eggs, not with anything so prosaic as a spoon, but with a "sprigg of rosemary or birch." Or she would, in the springtime, send the children into the fields, that she might prepare a store of "an excellent water for one that is in a consumption." Three pints of new milk, one pint of red wine, 24 new-laid eggs beaten together with as much white bread as would drink up the wine; then this was all distilled with cowslip flowers. And our ancestresses were fain to believe that one spoonful of it, taken morning and evening in chicken or mutton broth, would cure any consumption!

The last receipt to be quoted sounds more like a cannibal feast than a "gentlewoman's delight."

To make a warden pie. Bake your wardens with a little water, make a high coffin, and put them in whole!

But it was not so bloodthirsty in reality as in appearance; for a warden was, according to an old gardening authority, a sort of large pear commonly used for baking.

### American District Work.

BY A CORRESPONDENT.

While I was doing private nursing in a large American city I called upon the superintendent of the district nursing association to learn something of their methods of work. My experience in England was asked and a district offered me. This I could not accept, but was willing to help in any emergency.

A few days afterwards I was called to the telephone and requested by the superintendent to take work for one night. The patient, a baby, was not expected to live until the morning, and none of the staff could be spared to stay with it. I agreed to go, and was told to meet the doctor in attendance upon the case at 9 p.m. at a hotel near to where I was living, there to receive instructions and the address of the patient. The address given me was Pleasant Street, which I reached after much inquiry, to find that it was far from what its name implied, and at 9.30 on a sweltering July night it was simply alive with men, women, and children sitting and lying on the pavement and steps of the houses trying in vain to get a breath of air and coolness, before retiring to their hot rooms. I had some difficulty in finding the patient, as the name was that of a Russian Jew and was beyond my pronunciation. At length I succeeded, and was ushered into a cellar containing two rooms in which dwelt a whole family. The place was stifling. Now another difficulty arose; none of them spoke English, and as an American nurse does not wear uniform, the reason for my intrusion was not obvious. After various gesticulations one member of the family went out into the street and brought back another of their fraternity who spoke a little English. I then explained my presence and was allowed to take charge of the baby, a little girl, which I found in a mass of rags in a small cot. It was a puny, emaciated specimen of humanity, and would have been better in one of the beautiful hospitals which the city contains; but, as the family had been only a few months in the country, they would not trust it to the care of foreigners. The temperature was high, and the child was evidently suffering from broncho-pneumonia. I made her comfortable and sat down. My surroundings were too interesting for me to read or work—I had never seen anything quite like it in my nursing experience. The bed and everything in the room were absolutely filthy; the other children, two in number, lay without any covering but one small garment, which, I should judge, served both for day and night duty. The father of the family retired to the second room of the cellar, originally intended for the reception of coal, but which was now a kitchen. The mother, before settling down, proceeded to spread a meal, which was intended for me. I began to wonder how I should explain that I had dinner just before coming and required nothing more that night. I was saved any explanation, however; bananas formed part of the meal, and some of those I managed to eat.

It was not until early morning that I was trusted alone with the baby, but at last the worn-out mother fell asleep. With the light of dawn I found myself in even a worse predicament than I imagined, for the room was literally alive with vermin! I had never seen such a variety. My feelings can be better imagined than described. The weary hours wore away at last, and 8 a.m. brought the arrival of the doctor. He was surprised to find the baby still alive, and asked if I had any suggestions to make. I believed the child was dying from lack of fresh air, and asked permission to try the open-air treatment. It was granted, and together we carried the cot and baby up a number of flights of stairs and out on to a flat roof, and in a sheltered corner left it in charge of the mother.

I hurried home as quickly as possible to take my bath and change my clothes. As I was living with friends, I did not feel in justice to them that I could do another night of district work.

The superintendent telephoned me afterwards that the poor little one died five days after. The open-air treatment was evidently too late, but it probably prolonged life for a few days.

### Everybody's Opinion.

### THE AVERAGE COST OF DIET.

"Matron of a Fever Hospital" writes: It would be interesting and instructive to learn the experience of other matrons with regard to the daily menu for patients and staff in an infectious diseases hospital, where the average cost per head is 8½d. or 9½d. per diem. Whilst a patient is on milk only, no doubt he can be kept for 6d. daily or even less, but a scarlet fever patient who has to be on milk for any appreciable time will require extras. In the case of enteric patients the addition of ice, mineral waters, and stimulants in more than half the cases will also bring up the cost. I find that it costs 9s. or 9s. 6d. per week each for my staff. We get the majority of our provisions by contract, which in these days of keen competition seems to me the wisest and best course. It gives the matron a little more work, perhaps, and no doubt there are some tradesmen who may try to do you; but if they find that weights are checked, and goods not up to sample rejected, they very soon realise that it is their wisest course to be honest. With regard to committees, my opinion is that committees, at any rate of fever hospitals, are very much what matrons make them. Given a competent, straightforward matron, the majority of any committee will support her thoroughly. Of course, owing to the deplorable reduction in qualification for membership of local governing bodies of late years, there must of necessity, from time to time, be a small proportion of ignorant members prone to make insulting remarks and unfounded charges, but the intelligent majority will generally counteract their influence.

### NURSING IN A MILITARY HOSPITAL.

"One Who Should Know" writes, in answer to the letter from "A Kentish District Nurse": Having worked in military hospitals for over five years, I feel I would like to say that, in any hospital I have been in so far, it would be impossible for an orderly to make patients work in the manner described. To begin with, "a patient with a temperature of 105°, and suffering from pneumonia," would be so ill that it is very difficult for me to imagine him out of bed even, much less working. The supervision is now so strict that any orderly daring to be so brutal would soon find himself a prisoner. There is always a certain percentage of men coming into hospital with very little wrong, and who are, to use the soldier's slang, trying to "work their tickets," *i.e.*, leave the service. These men are allowed to do light duty, which means dusting, making their beds, and any other work the sisters or wardmasters think fit, but they are not told to do these things unless it has been marked on their diet sheets by their medical officer. Convalescent patients are treated the same according to the severity and length of their illness, and I usually find they are only too glad to help the orderlies in appreciation of all that has been done for them. If any patient thinks he is imposed upon in this respect, he has his remedy—namely, complain to his medical officer. The nursing service is so improved and so many more orderlies and nurses are now employed in most military hospitals, that the "light duty" is reduced to a minimum. The soldiers are well nursed and well fed when in hospital, and have very little to complain of nowadays, as quite a number of soldiers I know, who have been patients, could, and would, testify.

### Appointments.

**No charge is made for announcements under this head, and we are always glad to receive and publish appointments. The information, to insure accuracy, should be sent from the nurses themselves, and we cannot undertake to correct official announcements which may happen to be inaccurate. It is essential that in all cases the school of training should be given.]**

Ashton-under-Lyne District Infirmary.—Miss Laura C. Clifton has been appointed matron. She was trained at Westminster Hospital, and has since been assistant matron at the Western Hospital, Fulham, under the Metropolitan Asylums Board.

Barnsley Union Infirmary.—Miss Rhoda M. Hunt has been appointed superintendent nurse. She was trained at Birkenhead Union Infirmary, where she has since been sister. She holds the certificate of the Central Midwives Board.

Brompton Hospital Sanatorium, Frimley.—Miss Ida Mackintosh has been appointed matron. She was trained at the Sunderland General Infirmary, acted as district nurse in connection with the North London Nursing Association, and for some years as matron of the Royal Victoria Hospital, Ascot.

Children's Hospital, Bradford.—Miss Worthington and Miss MacPherson have been appointed sisters. Miss Worthington was trained at the Children's Hospital, Pendlebury, and Guy's Hospital, London, and holds certificates from the Central Midwives Board and from the Incorporated Society of Trained Masseuses. Miss MacPherson was trained at the Children's Hospital, Aberdeen, and has since been sister of the children's ward at Macclesfield General Infirmary.

Crewe Isolation Hospital.—Miss Mary Chrystie has been appointed sister. She was trained at the Royal Infirmary, Perth, and the Glasgow Maternity Hospital. She holds the certificate of the Central Midwives Board.

Gore Farm Hospital, Dartford.—Nurse S. Long has been appointed charge nurse. She was trained at St. Marylebone Infirmary, has since done private nursing at Eastbourne and Hastings.

Milton Hospital, Portsmouth.—Miss Mary Watkins has been appointed night sister. She was trained at Mill Road Infirmary, Liverpool, where she afterwards became ward sister. She has since been ward sister of the Bolton Borough Hospital.

Rochdale Infirmary.—Miss Ada Lee Thompson has been appointed night sister. She was trained at the Burton-on-Trent Infirmary, and has since been staff nurse at the Coventry and Warwickshire Hospital, and sister at the Halifax Royal Infirmary. She has also done district work near Windermere, and private nursing in London.

St. Anne's Home, Herne Bay.—Miss Florence Kite has been appointed charge nurse. She was trained at Greenwich Infirmary, and has since held the posts of ward nurse at Camberwell Infirmary, and superintendent nurse at Canterbury Infirmary, St. Olave's Workhouse, and the Trowbridge and Melksham Infirmary.

Southwark Infirmary.—Miss Eva Cameron Lowe has been appointed sister. She was trained at the Southwark Infirmary.

### Death in Our Ranks.

We regret to record the death of Nurse Mabel Maud Goodrunn, who died of tubercular disease, in May last, at the General Hospital, Johannesburg. She was trained at the General Hospital, Huddersfield.

The death of Miss Mary Ingham, superintendent of the Rochdale Nurses' Home, occurred recently. She has held her position for five years, and prior to that appointment she was a district nurse at Longridge, near Preston, for about six years. The funeral was attended by a large number of friends.

The many friends of Miss Charlotte Andrews will regret to hear of her death. Miss Andrews was trained at the Stapleton Union Hospital, Bristol, and was afterwards charge nurse for two and a half years. In August of last year she joined the "Lady Ampthill" Nursing Institute, leaving England in October for Madras, British India, where her death took place on August 16, from cholera.

### TRAVEL NOTES AND QUERIES.

By our Travel Correspondent.

Homes of Rest (Globule).—Your question does not belong to this column, but I have sent on your letter to the proper department.

Various Fares (Nemo).—First-class return to St. Malo, £2 12s. 8d.; second-class return, £1 19s. 6d. Climate much the same as ours on the south coast, but there is less rain and more sun. First-class return to San Remo, £11 9s. 3d.; second-class return, £8 4s. 3d. Climate usually mild and even warm in the winter, but warm clothes should be taken for occasional cold winds. First-class return to Rome, £15 14s. 11d.; second-class return, £11 4s. 9d. Climate in winter somewhat cold, but dry and clear, with a large proportion of sun. From what you tell me I think St. Malo would be quite unsuitable, and on the whole Rome seems best to suit your needs.

France or Belgium (Nurse).—I fear it is difficult to find anything at this season at your terms. Write to Mrs. Dyote, Villa Olga, Paramé, St. Malo, France, on a return prepaid letter-card and ask her if she has room for you and what are her lowest terms. She might take you at your highest figure. Why did you not write to me much earlier in the season, it is now so late. The journey to St. Malo, second-class return, is £1 19s. 6d. If this is too much, the fare to Knokke in Belgium is less: second-class return from Tower Bridge to Ostend, 9s., thence a short journey to Knokke. Try Hôtel de la Marine. I think you might be taken at 5 francs per day. If that house is full try the "Grand Hotel."

**Rules in Regard to Correspondence for this Section.—All questioners must use a pseudonym for publication, but the communication must also bear the writer's own name and address as well, which will be regarded as confidential. All such communications to be addressed "Travel Correspondent, 28 Southampton Street, Strand." No charge will be made for inserting and answering questions in the inquiry column, and all will be answered in rotation as space permits. If an answer by letter is required, a stamped and addressed envelope must be enclosed, together with 2s. 6d., which fee will be devoted to the objects of "The Hospital" Convalescent Fund. Ten days must be allowed before an answer can be published.**

### A Book and its Story.

NEW NOVEL BY IZA DUFFUS HARDY.*

"The Reason Why" is a slightly constructed but well-worked-out novel. The theme is the old one of lovers' misunderstandings, which in this case do not end happily. Readers experienced in the gentle art will have their doubts as to whether the two who parted so abruptly had not on either side sufficient cause to realise that two temperaments, which from social training and natural gifts were so diverse, could have found happiness in marriage at this period of their life. But the wisdom which is said to come with years, even in matters exempt from ordinary rules, came by voluntary separation to Jared Glasham and Kate Silvester. She, at least, being the weaker and more inexperienced, found to her sorrow that to rush out of one engagement from pique and fly into another from feelings difficult to define, wounded vanity, perhaps, being the most active factor in the proceeding, was to lay up a store of mental unrest which no amount of affection on the part of the second lover, whom she married, nor any fine acting on her own could allay.

"Bonnie Kate," as her friends called her, is rather a foolish, pretty, little girl, without money or any claims to distinction except those of youth and good looks and ambitions, which aspired chiefly to capturing a rich husband than to anything higher. Upon the horizon of the circle in which "Bonnie Kate" moves had arisen, when the story opens, a star of unusual magnitude in Jared Glasham, an American millionaire. He was a man who owned once, in reply to a query of hers of, "Are you never afraid of anything?" that fear was a word that was not in his dictionary; he did not even know what the feeling was. To Kate the assertion roused at once a feeling of interest in Glasham personally, apart from anything associated in her mind with his former life and surroundings. "In an Englishman—of her own world—the boast would have roused her derision; she would have laughed at it pitilessly as brag and swagger, but in this man, who came from a world that was strange to her, the cool, self-assertion did not repel her." She was attracted by the rugged strength, the directness of his speech, which seemed all a part of a personality utterly unlike that of any man she had met. A certain gravity of bearing also gave weight, and the Western habit of speaking without smiling added force to his remarks. Their first meeting was at a garden party on the banks of the Thames.

"The scene was all beauty, the day all brightness; and this English girl by his side might well have been the blossom of the bright day itself—a human flower, a beam of living sunshine." His impressions of Kate are summed up by mental comparison with girls of his own country in the following words: "Perhaps she was not as 'smart' and quick as an American girl; but she was sweet and fresh and frank and bright—and how pretty! with her soft, fluffy brown hair, in the ruffled waves of which the sunbeams seemed to be playing hide and seek, her eyes a darker brown, deeply fringed by curling black lashes, her clear-cut, delicately-moulded features, and dazzling fair complexion. While Glasham is making mental notes, Kate on her side is also reflecting on certain advantages of which he was the possessor. For instance—"Mr. Glasham might not be handsome, he might not be thoroughbred, he might have what seemed to her an objectionable American accent, he might come from another and a rougher way of life than hers, but he was rich—rumour ran wild in reporting how rich—and he was at present the biggest lion, the brightest rising star in the set which made up Kate's little world. . . . She cherished in her heart a secret admiration for strength, even if it were coarse, for courage, even if in its roughest form, of mere animal hardihood; and some instinct told her that it was no idle brag and baseless boast when this man said, so simply, that he did not know the feeling of fear. . . . There was, too, something not altogether unattractive about the somewhat hard-featured, bronzed face, with the heavy, square brow, the full, dark moustache, and the strong, close-cut crop of black hair, streaked by one or two threads of premature grey, for Jared Glasham was a good deal younger than he looked.

Glasham sustained an American's reputation for smartness in his wooing of Kate. He is not long in proposing to her. She accepts him with mingled feelings, but in spite of the prospect of the brilliant future which awaited her as a millionaire's wife, a very real feeling of affection for Glasham was a moving one. Unfortunately, all too soon, the little rift within the lute becomes perceptible. Glasham had been an unwilling listener to some bantering remarks about himself between Kate and her girl friends before his proposal, but the glamour which she had cast over him had made him risk the offer, in spite of the scene of which he had been an unobserved onlooker. Then, after their engagement was announced, he is again made aware of the possibility of Kate's acceptance being guided by the attraction of money and not by attachment to himself. "It was a habit at Silvermead to have the doors open in warm weather, and on this sunny afternoon the drawing-room door was open, a large Japanese screen shielding the ladies at the tea-table from all risks of a possible draught. They were mistaken in their comfortable conviction that they had the house entirely to themselves. One member of the party had returned earlier than they expected and passed the open door, his quick, quiet step falling unheard on the soft rugs whilst they were talking. . . . Harriet Silvester was just saying, in a tone of confidence, 'Judith Baldwin told me she always said she wasn't a bit in love with him.' 'Nobody ever supposed she was, unless indeed he's deluded enough to think so, poor man!' Mrs. Venables rejoined, with unnecessary acerbity 'she's in love with his banker's book.' Glasham, stung to the quick by these remarks, realising the gulf which socially, apart from the power of mere money, lay between Kate and himself, is filled with misgivings. 'Was she not too fair, too young, to love him?' he thought, with a twinge of jealous bitterness." Suffering from suppressed chagrin, he meets Kate, and in a scene which is very cleverly pourtrayed, the two, each animated by misleading thoughts, speak bitter words, which end in parting. In spite of this—"Never, perhaps, had the mutual attraction between these two been stronger; but the very strength of an attraction sometimes produces reactionary impulses, and in Jared Glasham at this hour it aroused a curious resentful and rancorous feeling. This soft, dainty creature, who seemed so fragile beside his rougher, coarser strength—so young and fair that people thought him a fool for dreaming that she could love him . . . and yet, there had begun to dawn a faint glimmer of feeling that Kate was fond of him after all. But it was such a tiny, feeble spark of faith—one little breath extinguished it."

As a character study Jared Glasham is particularly clever. It is a natural and consistent picture of a self made man launched into society and received and courted simply for his millions. Other characters are in a lesser degree well drawn, and the dialogue throughout the book is smart and to the point, when necessary, and always characteristic of the people who make it. "The Reason Why" is not a very happily-chosen title, perhaps, for a book which, in spite of some weak points, is, on the whole, very readable.

* "The Reason Why." By Iza Duffus Hardy. (Digby, Long, and Co.)

### Notes and Queries.

**REGULATIONS.**

**The Editor is always willing to answer in this column, without any fee, all reasonable questions, as soon as possible.**

**But the following rules must be carefully observed.**

**1. Every communication must be accompanied by the name and address of the writer.**

**2. The question must always bear upon nursing, directly or indirectly.**

**If an answer is required by letter a fee of half-a-crown must be enclosed with the note containing the inquiry.**

*Incurables.*

(168) Can you tell me of an institution where a man with cancer of the throat (incurable) is likely to be admitted on payment of about 12s. weekly?—*Mrs. P.*

Write to the Hon. Superintendent, Friedenheim Hospital, Upper Avenue Road, Swiss Cottage, London, N.W., or Free Home for the Dying, 29 North Side, Clapham Common, S.W.

(169) 1. Can you tell me of a free home into which I can get a child of four years old, suffering from rickets of the head, and being unable to talk? 2. Also the address of a home for the dying for a gentleman. The wife would pay a little?—*A Nurse.*

1. Write to the Secretary, Cheyne Hospital, Cheyne Walk. Chelsea, London, S.W., or Hospital for Incurable Children, North Court, Hampstead, N.W. (2) See reply to "*Mrs. P.*"

*Domestic Economy.*

(170) Will you kindly tell me where to write for information as to how I could get instruction in domestic economy, as I want to get a certificate for it?—*M. U. F.*

Write to Miss Romley Wright, Victoria Park, Manchester School of Domestic Economy, Manchester.

*Home.*

(171) I shall feel greatly obliged if you will let me know a home where an old lady of 76, who sometimes wanders in her mind, but is perfectly harmless, could find rest. Her relations would contribute about 10s. per week for her support, but as they themselves are poor they cannot do much.—*M. H. R.*

Write to St. Peter's Home and Sisterhood, Mortimer Road, Kilburn, London, N.W.

*Typhoid Crisis.*

(172) 1. When does the crisis in typhoid take place? 2. Should a new-born baby have a small dose of castor-oil soon after its birth?—*E. C.*

1. There is no crisis in typhoid fever. 2. Not as a matter of routine.

*America.*

(173) Will you kindly tell me if a mental nurse with experience in general nursing, but not trained, could get work in North-West America, and where I could apply for information of same?—*M. M. A.*

An untrained and uncertificated nurse would stand even less chance of success in America than in England. In any case we should not advise you to go there unless you have a certain amount of money in hand, introductions, and references.

*Training.*

(174) I should be grateful if you will tell me where I could find a hospital, institution, or infirmary (as near London as possible) where they would take me as a probationer; age between sixteen and seventeen.—*E. R.*

You are much too young to enter a general hospital, institution, or infirmary. When you are a little older you might be taken in a cottage hospital, children's hospital, or convalescent home.

*Tuberculosis.*

(175) Can you tell me if such a thing exists in England as a league for the prevention of the spread of tuberculosis and, if so, who is president? If there is such a league I am most anxious to know how one could get an insight into the work, being interested in a league about to be formed in Egypt for the same purpose?—*H. C.*

Write to the National Association for the Prevention of Tuberculosis, 20 Hanover Square, W.

**Handbooks for Nurses.**

| | Post Free. |
|---|---|
| "How to Become a Nurse: How and Where to Train." | 2s. 4d. |
| "Nursing: its Theory and Practice." (Lewis.) ... | 3s. 6d. |
| "Nurses' Pronouncing Dictionary of Medical Terms." ... | 2s. 0d. |
| "Complete Handbook of Midwifery." (Watson.) ... | 6s. 4d. |
| "Preparation for Operation in Private Houses." ... | 0s. 6d. |

**Of all booksellers or of the Scientific Press, Limited, 28 & 29 Southampton Street, Strand, London, W.C.**

### For Reading to the Sick.

PERSONAL SERVICE.

If any little word of mine
May make a life the brighter,
If any little song of mine
May make a heart the lighter,
God help me speak the little word,
And take my bit of singing
And drop it in some lonely vale,
To set the echoes ringing!

If any little love of mine
May make a life the sweeter,
If any little love of mine
May make a friend's the fleeter,
If any lift of mine may ease
The burden of another,
God give me love, and care, and strength,
To help my toiling brother!

*Anon.*

Others are affected by what I am, and say, and do. And these others have also their sphere of influence. So that a single act of mine may spread in widening circles through a nation or humanity.—*W. E. Channing.*

Let no one say that he must be better before he can take a part, even a quiet and unobserved part, in making others better. On the contrary, there is no duty which may not be made the gate of the road to Christ. Begin here and you will find that this way leads, where all such ways always lead, to your Master's presence. Do not fear that you are unworthy to serve Him, for serving Him in any way is the sure means to make you more worthy.—*Bishop Temple.*

We cannot always be doing a great work, but we can always be doing something that belongs to our condition. To be silent, to suffer, to pray when we cannot act, is acceptable to God. A disappointment, a contradiction, a harsh word, an annoyance, a wrong received and endured as in His presence, is worth more than a long prayer; and we do not lose time if we bear its loss with gentleness and patience, provided the loss was inevitable, and was not caused by our own fault.

*Fénélon.*

God makes every common thing serve, if thou wilt, to enlarge that capacity of bliss in His love. Not a prayer, not an act of faithfulness in your calling, not a self-denying or kind word or deed, done out of love for Himself; not a weariness or painfulness endured patiently, not a duty performed, not a temptation resisted, but it enlarges the whole soul for the endless capacity of the love of God.

*E. B. Pusey.*

Give thy heart's best treasures,
From fair nature learn;
Give thy best and ask not,
Seek not a return:
And the more thou givest
From thy little store,
With a double bounty
God will give thee more.

